# Apoptosis and DNA Methylation

**DOI:** 10.3390/cancers3021798

**Published:** 2011-04-01

**Authors:** Huan X. Meng, James A. Hackett, Colm Nestor, Donncha S. Dunican, Monika Madej, James P. Reddington, Sari Pennings, David J. Harrison, Richard R. Meehan

**Affiliations:** 1 MRC Human Genetics Unit, IGMM, Western General Hospital, Edinburgh EH4 2XU, UK; E-Mails: huan.meng@hgu.mrc.ac.uk (H.X.M.); jhackett@hgu.mrc.ac.uk (J.A.H.); colm.nestor@hgu.mrc.ac.uk (C.N.); donncha.dunican@hgu.mrc.ac.uk (D.S.D.); monika.madej@hgu.mrc.ac.uk (M.M.); james.reddington@hgu.mrc.ac.uk (J.P.R.); 2 Breakthrough Research Unit, University of Edinburgh, Western General Hospital, Edinburgh EH4 2XU, UK; E-Mail: david.harrison@ed.ac.uk (D.J.H.); 3 Queen's Medical Research Institute, University of Edinburgh, Edinburgh EH16 4TJ, UK; E-Mail: sari.pennings@ed.ac.uk (S.P.)

**Keywords:** epigenetics, Dnmt1, apoptosis, Mbd4, TDG, demethylation, 5-hydroxymethylation, DNA repair

## Abstract

Epigenetic mechanisms assist in maintaining gene expression patterns and cellular properties in developing and adult tissues. The molecular pathology of disease states frequently includes perturbation of DNA and histone methylation patterns, which can activate apoptotic pathways associated with maintenance of genome integrity. This perspective focuses on the pathways linking DNA methyltransferases and methyl-CpG binding proteins to apoptosis, and includes new bioinformatic analyses to characterize the evolutionary origin of two G/T mismatch-specific thymine DNA glycosylases, MBD4 and TDG.

## 1. Introduction

Epigenetics is concerned with alterations in phenotype caused by changes in cellular properties that are inherited but do not represent an alteration in genotype. In vertebrate somatic cells, epigenetic regulation of gene expression is thought to reinforce stable expression states at different loci. These are associated with particular molecular signatures of DNA and chromatin modifications connected with active and repressed chromatin states [1]. Epigenetic signatures such as DNA methylation are developmentally regulated and are thought to define comparable tissues and differentiation states [2]. In cancer, as well as in embryos generated through somatic cell nuclear transfer, normal patterns of DNA methylation are altered. These changes are thought to underlie the molecular pathology of these ‘disease’ states [3,4,5]. Not surprisingly, it has been proposed that there are signaling cascades that respond to perturbation of epigenetic regulatory mechanisms during development, which may ultimately result in activation of apoptotic pathways. In this short review, we will summarize the role and components of the epigenetic machinery, with an emphasis on DNA methylation, and their potential involvement in activating apoptotic pathways. In addition, we characterize the evolutionary origin of two G/T mismatch-specific thymine DNA glycosylases, MBD4 and TDG.

## 2. DNA Methylation

A major form of epigenetic information in mammals is carried by DNA methylation, which comes in the forms of 5-methylcytosine (5mc) and the more recently discovered 5-hydroxymethylcytosine (hmC) [6,7]. For 5mC, a methyl group is added covalently to the 5-position of cytosine by DNA cytosine methyltransferases (DNMT's), mostly within the context of CpG dinucleotides in somatic cells; however, non-CpG methylation also occurs at a high frequency in mouse and human embryonic stem (ES) cells [8,9]. Non-CpG methylation seems to be a feature of the pluripotent state, as it is present in induced Pluripotent Stem Cells (iPS) generated by transduction of a non-pluripotent somatic cell with stem cell-associated genes, which results in reprogramming of the recipient cell‘s epigenetic profile [8]. A recent discovery is the identification of a second modification in vertebrate DNA, hydroxymethylcytosine (hmC), in Purkinje neurons and embryonic stem cells [6,7]. hmC is formed by adding a methyl group to cytosine and subsequently an hydroxy group, in a reaction mediated by the Tet (Ten Eleven Translocation) family of enzymes [10,7]. Its importance in epigenetics is that the hydroxymethyl group is suggested to alter the biological properties of methylated DNA. It is worth noting that the methyl groups of both thymine and 5mC are susceptible to oxidation with 5mC being slightly more reactive resulting in the generation of hmC [11]. hmC also presents a new experimental problem, as conventional techniques (with the exception of a hmC specific antibody), cannot distinguish between 5mC and hmC in DNA [12,13]. Until this apparent anomaly is resolved, there will be some uncertainty in existing DNA methylation data bases. Recent technical developments can now distinguish prominent hmC sites in the genome [14]. However, it is clear that hmC is less abundant then 5mC, and the latter is still the most prominent modification in vertebrate DNA in many tissues. 5mC values are stable at a typical value of around 4.5% of all cytosine in tissues, whereas hmC values vary significantly [15]. This suggests that hmC has a specific function that is not absolutely correlated with 5mC levels, initial analysis suggests that hmC is predominantly associated with the gene bodies of highly expressed genes [14]. The replacement of a 5mC residue with hmC can inhibit the binding of methyl-CpG binding proteins (MeCPs) interfering with their functional roles in transcription [16].

## 3. The DNA Methylation Machinery

The presence of DNA methylation at regulatory sequences in somatic cells is generally associated with transcriptional repression and potentially has a long term impact on the stability of gene expression and on genome stability [17]. In humans, alterations in genomic methylation patterns are involved in the etiology of imprinting syndromes such as Beckwith-Wiedemann, Prader-Willi and Angelman, and have been implicated in a number of other disease conditions [18]. In mammals, the enzymes responsible for targeting and maintaining global DNA methylation are constructed from a complex set of functional modules, broadly divided into the N-terminal ‘regulatory’ domain and the *C*-terminal ‘catalytic’ domain. The regulatory domain functions largely as an interaction module, allowing multiple protein-protein interactions, DNA binding and nuclear localization [18]. Conversely, the *C*-terminal domain comprises ten motifs responsible for the enzyme's catalytic activity; six of these motifs are conserved in nearly all cytosine methyltransferases across the evolutionary spectrum from bacteria to mammals.

**Figure 1.**
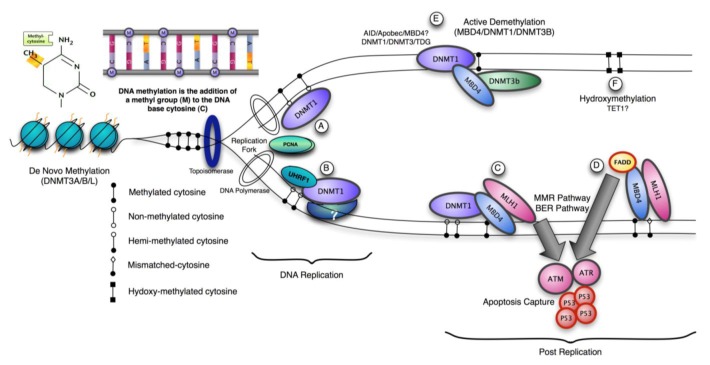
Maintainance of DNA Methylation. (A-B)The preferential substrate for the maintenance cytosine methyltransferase, DNMT1, is hemi-methylated CpG sites resulting from newly synthesized DNA in somatic cells. DNMT1 is present at the replication fork, and functions with the help of partner proteins including UHRF and PCNA. (C) Post-replication, DNMT1 and a methyl-CpG binding protein MBD4 can be localized together at DNA damage sites and may be part of cellular pathway response that activates apoptosis. MBD4 interacts directly with both DNMT1 and MLH1 leading to recruitment of all three at DNA damage sites. (D) MBD4 has also been shown to recruit Fas-associated death domain protein (FADD), which bridges death receptors with initiator caspases. FADD may also be an apoptotic effector via MBD4. (E) Several active DNA demethylation models have been proposed. MBD4 was reported to execute active DNA demethylation at the CYP27B1 promoter in response to PTH (Parathyroid Hormone) signaling. Similarly TDG (Thymine DNA glycosylase) can also interact with Dnmt3A and Dnmt3B and function as 5meC glycosylase activity against hemi-methylated DNA with the same weak excision activity as MBD4. In zebrafish embryos Aid, Mbd4 and the DNA repair protein Gadd45a may cooperate to induce demethylation. Thymine glycosylases such as TDG and MBD4 may function on deamination of 5-methyl-cytosine by repairing the resulting mismatch. (F) TET1 is capable of acting on both fully methylated and hemi-methylated DNA, producing 5-hydroxymethylcytosine (5hmC) in DNA, which may also act in signaling pathways associated with turnover and maintenance of the epigenome.

Three methyltransferase enzymes, Dnmt1, Dnmt3a and Dnmt3b coordinate the establishment and maintenance of DNA methylation patterns in mammals (Figure 1). The ‘*de novo*’ methyltransferases, Dnmt3a and Dnmt3b, target cytosine methylation to previously unmethylated CpG dinucleotides, whereas the ‘maintenance’ enzyme, DNA methyltransferase-1 (Dnmt1), preserves existing methylated sites [18]. DNMT3a and 3b are thought to be *de novo* methylases with an equal preference for hemimethylated and unmethylated DNA, which are necessary for *de novo* methylation of the genome during development and potentially newly integrated retroviral sequences [19,20]. The *N*-terminal region interacts with many chromatin-associated proteins including the *de novo* methyltransferases, MeCPs and histone modifying enzymes. It also contains a nuclear localization signal, a PCNA (proliferating cell nuclear antigen)-interacting domain, a replication targeting region and a cysteine-rich Zn^2+^-binding domain that can potentially bind non-methylated CG rich DNA. DNMT1 also contains a domain showing homology to the polybromo-1 protein and is thought to mediate protein-protein interactions. Many of these interactions are involved in transcriptional repression [21,22]. Recent structural analysis suggests an elegant model for the maintenance DNA methylation function of Dnmt1 in which its CXXC domain specifically binds to unmethylated CpG containing DNA and resulting in a repositioning of the CXXC-BAH1 linker between the DNA and the active site of DNMT1, preventing *de novo* methylation [23]. Furthermore, a loop projecting from BAH2 domain interacts with the target recognition domain (TRD), stabilizing it in a retracted position, and preventing it from accessing the DNA major groove. In consequence only hemimethylated CpG dinucleotides that do not bind the CXXC domain can gain access to the active site. The multiple interactions of DNMT1 suggest that it can be a participant in multiple complex networks involved in gene regulation, epigenetic signalling and genome stability. DNMT1 is also post-translationally modified and this can modulate its protein-protein interactions, protein-DNA interactions, subcellular localization, catalytic activity and its stability [24-26]. The protein lysine methyltransferase SET7 regulates DNMT1 activity in mammalian cells by promoting degradation of DNMT1 and thus allows epigenetic changes via DNA demethylation [27]. This modification on Lysine 142 is mutually exclusive with phosphorylation on Ser143; phosphorylated DNMT1 is more stable than methylated DNMT1 [28].

The respective *N*-terminal domains of Dnmt3a and 3b are responsible for their targeting to chromatin via a PWWP domain, and impart differences in specificity. In this respect, it appears Dnmt3b is specialised in methylation of specific regions of the genome, such as pericentromeric repeats and CpG islands on the inactive X-chromosome, whereas Dnmt3a is required for maternal imprints of differentially methylated regions (DMRs), in addition to their general *de novo* roles [29]. The PWWP domain of Dnmt3a specifically recognizes the histone 3 lysine 36 trimethylation mark and this may important for its subnuclear localization [30]. This interaction may underlie co-distribution of DNA methylation and H3K36me3 in chromatin [31]. Mouse embryos become hypomethylated during early development; reestablishment of global methylation depends on the *de novo* activity of Dnmt3a and Dnmt3b [20]. Once established, DNA methylation patterns are stably maintained over cell divisions by Dnmt1 through its preference for hemimethylated DNA. In addition deletion of Dnmt3a in primordial germ cells disrupts paternal and maternal imprinting, where as Dnmt3b is dispensable for mouse gametogenesis and imprinting [32,33]. A rare chromosome breakage disease called the immunodeficiency, centromeric region instability, and facial anomalies syndrome (ICF) is associated with point mutations in the DNMT3B gene leading to hypomethylation of satellite DNA, this hypomethylation phenotype can be recapitulated in a mouse model [34,35].

Protein interaction domains in the regulatory N-termini of Dnmt3a and Dnmt3b also mediate binding to transcriptional co-repressors [36]. Dnmt3b is associated with Sin3a, SUMO1/Ubc9, condensin and the chromatin remodeling enzyme hSNF2H, while Dnmt3a has been shown to bind the co-repressor RP58 and the oncogenic factor PML-RAR [18]. Both also interact with DNMT1, histone deacetylases (HDAC) and multiple transcription factors [37]. Thus, the cooperative function between DNMTs provides a way of passing on and maintaining epigenetic information between successive cell generations and this is reinforced through interaction with chromatin modifiers. Unlike DNMT1 and DNMT3A/B, the DNA methyltransferase DNMT2 has only weak methyltransferase activity *in vitro*, and its absence causes no discernable effects in global CpG methylation levels nor results in a developmental phenotype [18]. A cofactor, DNMT3L (DNMT3-Like), is expressed only in germ and ES cells. It is not a methyltransferase but enhances *de novo* methyl transferase activity in ES cells [38].

In addition to a role in gene silencing, 5mC is a prominent cause of mutations as its mutation to thymine (T) is 10–50-fold higher than other transitions [39]. Both Dnmt3a and 3b can interact with repair enzymes, thymine DNA glycosylase (TDG) and methyl-CpG binding domain 4 (MBD4), that have evolved to counteract the mutagenic effect of methylcytosines [40,41,42]. This implies a mechanistic link between DNA repair at sites affected by 5mC deamination and subsequent remethylation.

## 4. Role of DNA Methylation

The presence of DNA methylation (5mC) at CG rich promoters clearly acts as a deterrent to transcription. Known targets include imprinted genes, transposons, the XIST gene and CpG islands (CGIs) genes on the inactive X-chromosome. However, the extent to which promoter methylation regulates developmental and tissue specific gene expression is unresolved. In general, repression by DNA methylation is considered to occur downstream of other epigenetic or trans-acting factors that signal the initial inactivation event. For example, in the case of the pluripotency factor Oct3/4, initial repression during differentiation is mediated by sequence-specific repressors such as GCNF followed by conversion from an active to an inactive histone modification signature and lastly by the deposition of DNA methylation at the promoter [43,44]. Genome wide sequencing analysis suggests that the number of potential genes that can be directly regulated by DNA methylation in a tissue and developmental specific manner may be quite small corresponding to 100–200 of annotated CGI's in somatic cells [45]. However this may be an underestimate, as new data suggests there are approximately 23,000 and 25,500 CGIs in the mouse and human genomes respectively, about half of which are associated with annotated transcription start sites for mainly constitutively expressed genes [5]. As with previous estimates, around 2.5% of the annotated CGI's are methylated in somatic tissues and may be directly regulated by DNA methylation. In contrast the non-annotated or ‘orphan’ CGI's show higher levels of tissue specific methylation (14-20%) and lack marks of active transcription; histone H3 tri-methylation on lysine 4 (H3K4me3) and RNA polymerase II occupancy [5]. These may be directly regulated by DNA methylation in different tissues and developmental stages. The importance of the preservation of these patterns is suggested by the abnormal silencing of annotated tumor suppressor genes by unscheduled *de novo* methylation of their promoter CGIs, which can contribute to unchecked proliferation in neoplastic cells [17]. In addition, tumor-specific CGI methylation differed from that in normal tissues by not being preferentially targeted to orphans CGIs [5]. At the same time as *de novo* methylation of CGIs, global methylation levels associated with satellite repeats and retroposons are reduced in cancers. This may permit activation of typically silent transposons and contribute to genomic instability through illegitimate recombination events [17].

## 5. RNA Interference Pathway

RNA interference is a conserved process by which sequence-specific double-stranded RNA is converted into small interfering double-stranded RNAs (siRNAs) that can induce gene silencing via two pathways: post-transcriptional gene silencing and transcriptional gene silencing (TGS) [46,47]. TGS is well documented in plants but less so in mammals [47]. An initial report promoter targeted DNA methylation by siRNAs was subsequently retracted [48,49]. However it has been demonstrated that promoter- targeted siRNAs can induce silencing of simian immunodeficiency virus (SIV) replication by induction of methylation at a CpG site within the SIV promoter region following siRNA-induced suppression [50,51]. Different classes of small RNAs differ in their origin, biogenesis, expression pattern, and utilization. MicroRNAs (miRNA) are single-stranded RNA molecules aprroximately 21-23 nucleotides in length that can silence by binding to target mRNAs. It has been suggested that miRNAs are also directly involved in the maintenance of genomic integrity through global repression of transposable elements (TEs) and indirectly through regulation of DNA and histone modifying enzymes [52,53]. Interestingly, a noncoding RNA has been identified that interacts with the ribosomal DNA promoter in mouse NIH3T3 cells that mediates recruitment of Dnmt3b and subsequent silencing of ribosomal RNA genes [54].

## 6. Histone Modifications and Repression

While DNA methylation plays an important part in overall transcriptional repression, it is clear that animal cells also utilize additional networks to mediate gene silencing. In ES cells that lack all three active DNA methyltransferases, a relatively small percentage of genes become reactivated [55]. This contrasts with inhibition of Dnmt1 function a somatic cell [56]. Eukaryotic DNA is packaged with histone and non-histone proteins into chromatin and this has two important consequences. Firstly, it allows the packaging and compaction of ∼1.8 m of DNA into a nucleus typically 5-20 μm in diameter. Secondly, it promotes the regulation of essential cellular events including transcription, lineage specification, DNA replication and cell division. The fundamental unit of chromatin is the nucleosome, which is composed of an octamer of the four core histones (H2A, H2B, H3 and H4) around which 147 bp of DNA is super-helically coiled 1.65 times [57]. The repressive effect of nucleosomes on transcription can be enhanced or reduced by combinations of histone modifications [58,59]. The major effect of histone modifications is to regulate transcription by creating a docking site for non-histone effector proteins to modify chromatin structure. However in some cases, strongly charged modifications can alter chromatin structure directly, through disrupting the DNA-histone interaction. A diverse range of histone modifications have been reported including: acetylation, methylation, phosphorylation, ubiquitination, sumoylation, formylation, deimination, ADP ribosylation, and proline isomerization. Covalent post-translational modifications to the tails of histone proteins can reversibly affect gene expression by modifying their interaction with DNA and other nuclear proteins. These modifications can have either activating or repressive effects on the expression of surrounding genes, depending upon which histone residue receives the particular modification. The combination of these modifications and the resulting effect on gene expression is referred to as the “Histone Code” [60].

For example, trimethylation of lysine 4 on histone H3 (H3K4me3) is enriched at transcriptionally active gene promoters, whereas trimethylation of H3K9 (H3K9me3) and H3K27 (H3K27me3) is present at gene promoters that are transcriptionally repressed. The latter two modifications together constitute the two main silencing mechanisms in mammalian cells, H3K9me3 working in concert with DNA methylation and H3K27me3 largely working exclusive of DNA methylation. Genome-wide studies showing distinct localization and combinatorial patterns of these histone marks in the genome have significantly increased our understanding of how these diverse modifications act in a cooperative manner to regulate global gene expression patterns [17]. The polycomb complex (PRC), which mediates tri-methylation of lysine 27 on histone H3 (H3K27me3) appears to be targeted specifically to genes involved in development and differentiation [61]. DNA methylation and specific histone modifications can also influence each other during mammalian development and it is noteworthy that many of the components from each system interact such that histone H3 methylation at lysine 9 (H3K9me2/3) can help to direct DNA methylation patterns, and DNA methylation might serve as a template for some histone modifications after DNA replication [43,58]. Heterochromatin protein (HP1) binds H3K9me2/3 containing chromatin through its chromodomain and at the same time acts like a nuclear ‘Velcro’ protein through multiple interactions with protein partners via its chromoshadow domain [62]. H3K9me2/3 is generated by a multitude of histone methyltransferases including Suvar39H1, G9a and GLP. Interaction partners for HP1 include DNMT1, Suvar39H1 and G9a [21,22]. HP1 can inhibition preinitiation complex (PIC) assembly *in vitro* by blocking key subunits of the TFIID and Mediator coactivator complexes. Notably, binding of HP1 inhibited the Sp1-regulated survivin gene *in vivo* upon DNA damage-induced silencing [63]. Loss of HP1 proteins causes chromosome segregation defects and lethality in some organisms and a reduction in levels of HP1 family members is associated with cancer progression in humans [62].

In contrast histone H3 methylation at lysine 27 may be mutually exclusive with the presence of DNA methylation [64]. Genes targeted by polycomb actually undergo repression through a process of heterochromatinization. Binding of the PRC2 complex to specific genes brings about local tri-methylation of histone H3K27 by means of the histone methyltransferase Ezh2 contained in these complexes [65]. These methyl groups then serve as a ligand for the chromodomain protein, Cbx2, that is part of the PRC1 complex, and this generates a heterochromatin-like structure and gene repression. However polycomb target genes in ES cells can have a bivalent chromatin signature, being marked by both H3K27me3 and the activating modification H3K4me3 [1,61]. As development proceeds, the polycomb complex is removed in a gene and cell-type specific manner, thus activating or maintaining the silencing of those genes as required through development [66,61]. Like DNA methylation, gene silencing via histone modification can be maintained *in vivo* through multiple cell divisions. One possibility is that this occurs through the simple rebinding of repressor molecules following DNA replication.

It has been reported that CGIs that are aberrantly methylated in neoplastic cells coincide with sites targeted by polycomb in human ES cells [67]. Approximately 16% of all CGIs are H3K27me3 positive in human ES cells and these sites are not over-represented among the CGIs methylated in blood, cerebellum or normal colon [5]. In contrast, 56% of tumor-specifically methylated CGIs are derived from CGIs that were H3K27 trimethylated in embryonic cells [5]. These findings emphasise the distinction between tumor-specific CGI methylation and that found in normally developing human somatic tissues and reinforce the illegitimate link between polycomb complexes and tumor-specific CGI methylation. Loss of the Swi/Snf chromatin remodeler component SNF5 in malignant rhabdoid tumors leads to elevated expression of EZH2 [68]. Consequently, Polycomb targets are broadly H3K27-trimethylated and repressed in SNF5-deficient fibroblasts and cancers. Normally there is antagonism between SNF5 and EZH2 in the regulation of stem cell-associated programs so that Snf5 loss deregulates these programs.

## 7. Apoptotic Cellular Response to Epigenetic Perturbation

The utilization of epigenetic regulatory mechanisms in disease states can contribute to the molecular pathology by altering gene expression states that support progression and inhibit defense pathways such as tumor suppressor genes. For example, epigenetic inactivation of hMLH1 is a major cause of microsatellite instability in sporadic colorectal cancers, likewise epigenetic alteration of genes involved in the induction of senescence is often associated with cancers showing mutations in the Ras signaling pathway [69]. Hypomethylation of short and long interspersed repetitive elements has been reported in cancer, and hypomethylation of the genome has been observed in ICF syndrome [16,70]. Since epigenetic mechanisms are deeply embedded in regulating developmental gene expression programs, it is not surprising that loss of components of the epigenetic toolbox during development results in severely impaired or embryonic lethal phenotype. The cellular response is not governed just by transcriptome changes but also specific activation of signaling cascades that usually respond to DNA damage.

Despite obvious differences in biology and early developmental strategies, inhibition of DNMT1 function *Xenopus laevis* embryos shows remarkable similarity with mouse dnmt1^−/−^ mutants as indicated by the presence of axial defects, failure to form a neural tube, and improper patterning of the somites [71-73]. Underlying the phenotype in both species is activation of p53-mediated apoptosis [56,74]. One generalization from the analysis of Dnmt1 depletion or inhibition is that it is not essential for the survival of embryonic cells during early cleavage stages. Instead the effect of disrupting Dnmt1 function only becomes apparent during and past gastrulation when loss of Dnmt1 provides a signal through p53, to initiate apoptosis.

Recent work demonstrates that complete inactivation of DNMT1 function in human cancer cells results in cell death, but this decrease in viability occurs with minimal changes in global DNA methylation [75]. This observation supports the hypothesis that DNMT1 possesses essential functions independent of its role as a maintenance methyltransferase and links its absence with activation of a cellular checkpoint response. Partial reduction in xDnmt1p levels without changes in DNA methylation levels is sufficient to activate a cell death program in *Xenopus* embryos [76]. This contrasts with dnmt1^−/−^ ES cells, which proliferate normally in culture unless they are induced to differentiate, but notably they exhibit high rates of micro-satellite instability and DNMT1 (but not the *de novo* methyltransferases) can be recruited to sites of DNA damage via PCNA [77,78]. A possible mechanism for DNMT1 recruitment and signaling apoptosis is through the mismatch repair pathway (MMR). The methyl-CpG binding protein, MBD4 (MED1) has been shown to function as a thymine glycosylase and interacts with the MMR protein MLH1. An important function of MMR proteins is to sense DNA damage and mediate the decision to repair the lesion or to induce apoptosis in somatic cells [79]. The levels of several MMR proteins are reduced in mbd4^−/−^ mouse embryonic fibroblasts, which can account for the diminished apoptotic response of these cells to DNA damaging agents [80,81]. In wild type cells, DNA-damage recognition by MMR factors is sufficient to trigger cell-cycle arrest and apoptosis through direct interaction with signaling kinases such as ATM, ATR, CHK1, CHK2, which ultimately activates p53 [82]. MBD4 can interact directly with both DNMT1 and MLH1 leading to recruitment of all three components at DNA damage sites [83]. The co-localization of DNMT1, MBD4 and MLH1 suggests that they may participate in a cellular checkpoint that monitors potential DNA hypomethylation events by detecting the presence or absence of the maintenance methyltransferase, perhaps at or adjacent to the replication fork. The recruitment of these components in response to localized DNA damage suggests that can have a role in the cellular decision whether to repair the lesion or activate apoptosis [83]. Double depletion experiments in *Xenopus laevis* suggest that both MBD4 and MLH1 are required for the embryonic lethal phenotype of associated with DNMT1 depletion. Inhibition of p53, MBD4 or MLH1 alleviates phenotypic consequences DNMT1 inactivation in frogs. This suggests in this model system that it is the absence of DNMT1 at cellular checkpoint that triggers apoptosis by releasing MLH1/MBD4 to activate downstream DNA damage kinases such as ATM, which activate p53 and subsequent apoptosis. In agreement with this, over-expression of either MLH1 or MBD4 in MEFs resulted in activation of an apoptotic response and the glycosylase catalytic activity of MBD4 is not required for this [83]. In contrast, over-expression of MBD4 did not induce apoptosis in HCT116 cells, which lack functional MLH1, whereas over-expression of GFP-MLH1 did. This model suggests that the maintenance of genome integrity through DNA repair mechanisms includes preservation of epigenetic signatures, requiring the participation of DNA and histone modifying enzymes in repair pathways.

A recent report suggests that DNA methyltransferases are essential for maintaining the memory of a genotoxic insult that persists after exposure [84]. Mouse embryonic stem cells exposed to γ-radiation harbour the effects of the insult for weeks and conditioned media from the progeny of exposed cells can induce DNA damage and homologous recombination in naive cells. Experiments in ES cells suggest that the molecular pathway that underlies the memory of the insult requires Dnmt1 and Dnmt3a but not Dnmt3b [84]. These exciting data suggests a potential molecular pathway for persistent bystander effects associated with exposing cells to of DNA-damaging agents.

Ubiquitin-like, containing PHD and RING finger domains, 1, also known as UHRF1 or Np95, binds methylated CpG through its SET and RING finger-associated (SRA) domain [85]. It localizes to replicating heterochromatin and is dependent on the presence of hemi-methylated DNA. Np95 forms complexes with Dnmt1 and is essential *in vivo* to maintain global and local DNA methylation. Np95^−/−^ embryos exhibited growth retardation and various malformations such as neural tube closure defect, small branchial arches and failure in outgrowth of allantois. Like DNMT1/- embryos they exhibit excess apoptosis that may be independent of the transcriptional changes associated with DNA hypomethylation.

Like DNMTs, inactivation of many histone modifying enzymes in ES cells is not cell lethal but is detrimental to embryo development. The H3K9 methyltransferases G9a, GLP and Suvar39h1/2 are required for early embryo development, although mortality of G9a and GLP deficient embryos occurs earlier (by E8.5) than Suvar39h1/2 mutants (E14.5) [86,87,88]. The G9a and GLP mutants are very similar and TUNEL assays indicate that abundant apoptosis occurs in G9a^−/−^ embryos, whereas very few cells were positive in wild-type embryos [87]. The mechanistic basis of this apoptotic activation is unclear, however G9a derived MEFs are viable despite having an extensive gene mis-expression profile, which suggests there is a developmental specific checkpoint (as for DNMT1) that is activated upon G9a inactivation in early embryos [89]. By contrast, Suv39h double mutants exhibited genomic instability in a subpopulation of cells, which did not trigger extensive apoptosis [86]. This suggest that repeat DNA associated Suv39h-dependent H3-K9 methylation is important for maintaining a stringent higher-order structure at pericentric heterochromatin, which is required to protect genomic stability. Absence of Suv39h HMTase does not trigger cell death pathways. Similarly loss of the Polycomb components has not been reported to trigger apoptosis [65]. However, the S-adenosylhomocysteine hydrolase inhibitor 3-Deazaneplanocin A (DZNep) induces efficient apoptotic cell death in cancer cells but not in normal cells by depleting cellular levels of the PRC2 components EZH2, SUZ12, and EED [90]. This reduced H3K27me2/3 levels but not H3K9me2/3. In this case preferential reactivation of a novel apoptosis effector, FBXO32, by DZNep, may be responsible for inducing apoptosis in the cancer context. This would be consistent with a cancer specific phenotype in which epigenetic mechanisms have been co-opted to selectively repress genes that are antagonistic to transformation either by inhibiting cellular proliferation and promoting either senescence or apoptosis [91].

## 8. MBD4, Apoptosis and Mis-match Repair

**Figure 2.**
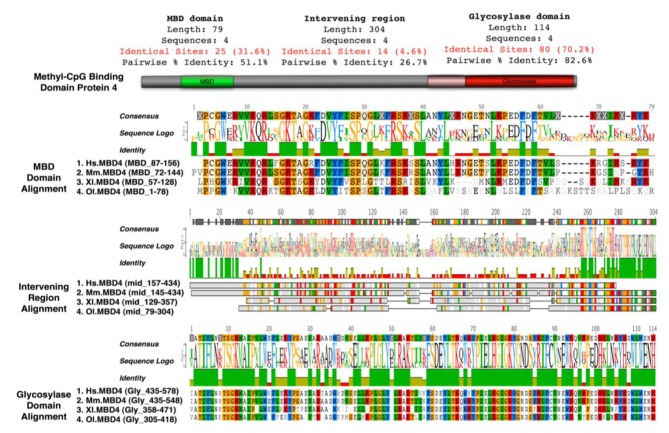
Functional domains of Methyl-CpG binding domain protein 4 (MBD4). A schematic highlighting the two domains of MBD4; an N-terminal MBD domain that binds methyl-CpG containing duplexes including mis-matches and a C-terminal Glycosylase domain, which can excise and repair both thymine and uracil mutations that result from deamination of methyl-cytosine and unmethylated cytosine. The glycosylase domain of MBD4 is well conserved, about 114 amino acids long, sharing 70.2% identical sites between species from Medaka Fish to Human. In contrast, the MBD domain of MBD4 has poor conservation, the 70 amino acids that constitute MBD domain sharing only 31.6% identical sites. The intervening region is a domain desert and highly variable in sequence. It is worth noting that a 50 amino acid region right ahead of the glycosylase domain is conserved between species. This region interacts with several important protein partners, including MLH1and FADD. Sequence alignments of the MBD domain, the intervening region and the glycosylase domain of MBD4 protein were generated with clustalX module of Geneious pro software.

**Figure 3.**
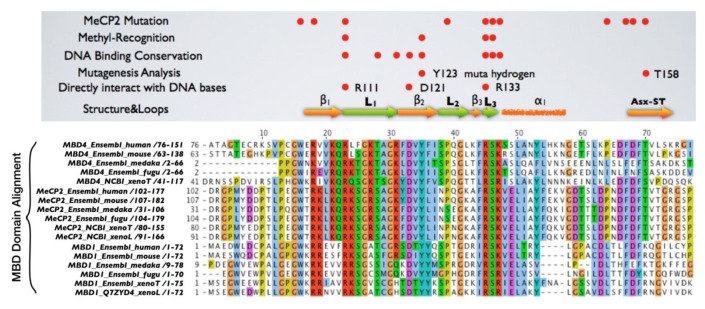
Key functional and structural amino acids are well conserved within MBD domain of MBD4. The MBD domain alignment is shown. A solution structure of the MBDs from MeCP2 and MBD1 has been determined, consisting of four anti-parallel β-strands, two of which were proposed to interact with the major groove of DNA, where a methyl group would be located. In addition, a number of conserved residues throughout the MBD domains of MeCP2 and MBD1 can be easily revealed by alignment, despite their full-length sequences sharing only moderate homology. The MBDs of MBD4, MBD1 and MeCP2 were aligned and compared to indicate essential residues within the MBD of MBD4 that are responsible for binding to methylated DNA sequences. Essential Residues are well conserved in the MBD of MBD4 [93]. In addition, amino acids within the MBD of MBD4 that are important for DNA binding found by mutational analyses and associated with Rett syndrome in MeCP2 are also well conserved. Sequence alignments of the MBD domain of MBD proteins were generated with clustalX module of Jalview software.

Mice lacking methyl-CpG binding proteins are viable during early development. With the exception of mbd4^−/−^ mice, there is no association with activation or impaired apoptotic signaling pathways [92]. MBD4 is a methyl-CpG binding DNA protein containing a highly conserved glycosylase domain at the C-terminal [1]. It is proposed to be involved in the repair of mismatches resulting from cytosine deamination. In contrast to the poorly conserved N-terminal MBD domain, the key amino acids that are responsible for binding specificity and structure are well conserved [92,93] (Figure 2 and Figure 3.) Spontaneous hydrolytic deamination of methylated cytosine causes C•T transitions at meCpG, and non-methylated CpG mutates to UpG. MBD4 was shown to excise and repair both C•T and C•U mutations at methylated and non-methylated CpGs via its glycosylase domain and adjacent binding site (Figure 2 and Figure 3). Novel interacting partners of MBD4 include MLH1 and Fas-associated death domain (FADD) proteins, suggesting a potential link between genome surveillance and apoptosis [94,95]. Consistent with these observations, reduced apoptosis occurs in the small intestine of mbd4^−/−^ mice in response to a variety of DNA-damaging agents, and increased tumorigenicity was observed for mbd4^−/−^ mice on a tumor-susceptible Apc min background [40,96,97]. Recently it has been demonstrated that TDG is essential for early mouse development and the embryonic lethal phenotype includes mis-expression of developmental genes suggesting it may have a structural role in maintaining sites of active gene expression [98]. There was some evidence that TDG might also function to erase aberrant methylation at normally methylation free CpG island promoters. A bisulfite deep sequencing approach of TDG mutant embryos may address this possibility more fully, with the caveat that the sites may be 5-hydroxymethylated and not 5mC modified.

It has been proposed that MBD4 proteins arose as a fusion protein between MBD and glycosylase domain ancestors in the vertebrate lineage [2]. MBD2/3 represents the ancestral methyl-CpG binding protein. Interestingly, a prototypical MBD4 protein and its putative ancestor MBD2/3 were both identified outside vertebrates in the cephalochordate amphioxus (*Branchiostoma floridae*) [99]. A putative MBD4 was predicted in the *Ciona intestinalis* genome, but it lacks an MBD domain. The finding of a putative MBD4 protein in this invertebrate-vertebrate transition model organism pushes back the origin of MBD4 proteins in evolutionary time [3]. (Figure 3) The MBD domain of MBD4 is most similar to that of MeCP2 in primary sequence, and binds specifically to methylated DNA [100,101]. MeCP2 has been described in several molecular roles including transcriptional repression, activation of transcription, nuclear organization, and splicing, whereas the role of MBD4 remains largely unknown apart from functions in DNA repair and apoptosis [92]. Noticeably, the primary sequence of the MBD domains of MBD4 proteins in vertebrates diverges much more than those of MeCP2 proteins, even though important residues that are responsible for DNA binding and Rett syndrome mutations etc. are well conserved (Figure 2 and Figure 3).

MBD4 gene mutations were found in tumors that exhibit genomic instability associated with defective DNA mismatch repair (MMR); termed primary microsatellite-instability (MSI). A characteristic of MSI tumors is frequent silencing of MLH1 and down-regulation of MMR target genes, such as MRE11 and MBD4 [102]. The somatic MBD4 mutations in these tissues are likely a consequence of MMR deficiency. Accumulated evidences have shown that MBD4 and several MMR proteins fall in the same break excision repair (BER) pathway. Interestingly, steady state amounts of several MMR proteins were found to be downregulated in mbd4^−/−^ MEFs [80]. Regarding tumor prognosis, reduced expression of MBD4 correlated with poorly differentiated tumors in hepatocarcinomas (HCC) [103]. In an investigation using 24, paired colorectal cancer samples, MBD4 was found to be a significant prognostic factor for patient survival by Kaplan-Meier survival analysis [104]. In addition, expression levels of MBD4 and MBD3 correlated with the grade of malignancy in human gliomas both *in vivo* and *in vitro* [105]. Thus the clinical relevance of MBD4 expression may be due to a defect in DNA damage signaling and repair pathways.

On a MMR defective background MBD4 mutation (due to polyadenine tract alterations) occurs frequently in human cancers leading to premature truncation of the MBD4 protein, which lacks the whole glycosylase domain [106]. It has been suggested that this truncated form of MBD4 acts in a dominant negative way, competitively inhibiting normal glycosylase activity of wild type MBD4, and increasing the mutation frequency when over-expressed in cells [107-109]. Inhibition of TDG (thymine DNA glycosylase) activity m5CG/GIU by the MBD domain was not observed [110]. This indicates the MBD domain possess an additional role in at least regulating MBD4 glycosylase activity, in addition to binding meCpGs and mismatched sites.

**Figure 4.**
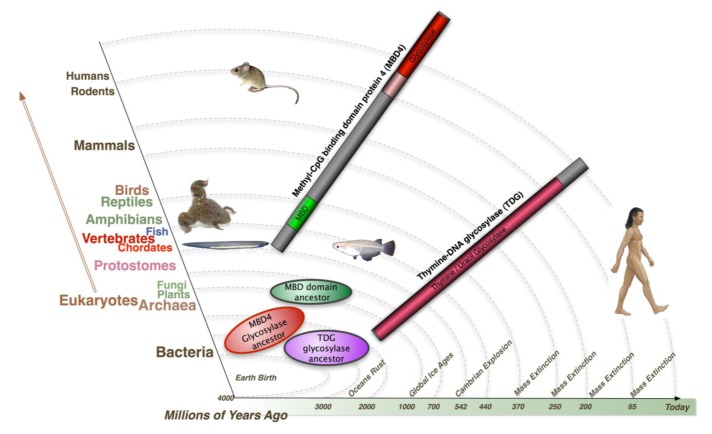
Evolutionary timeline of the two mammalian Thymine glycosylase proteins: Methyl-CpG binding domain protein 4 and Thymine-DNA glycosylase (TDG). The ortholog of proteins including the MBD domain and the glycosylase domain of human MBD4 and of full length human TDG were inferred initially by reciprocal best BLASTP searches against the non-redundant protein sequences database, and confirmed by phylogenetic reconstructions. Neighbor-joining (NJ) phylogenetic trees were constructed for domains of MBD4 and full length TDG proteins. Maximum likelihood (ML) analyses were performed using the PHYML module from Geneious pro software, following the JTT model of amino acid substitution. Protein domains were identified using InterProScan and Conserved Domains servers. Published procedures for this bioinformatic approach were followed [99]. Both glycosylase ancestors of MBD4 and TDG were found in Bacteria. Glycosylase ancestors of MBD4 were found in Archaea, whereas only a hypothetical TDG Archaea ortholog was identified. Both glycosylase ancestors of MBD4 and TDG were found in Plants and Fungi, in contrast, the earliest MBD domain ancestors were in plants and not in Fungi. TDG protein was found in almost all the species throughout evolution, the full length MBD4 though, emerges as a fusion protein only from Chordates: the representative of Invertebrate-Vertebrate transition.

Both TDG and MBD4 have been shown to be capable of removing guanine (G):uracil (U) mispair and guanine (G):thymine (T) mispair, products from deamination of the exocyclic amino group in cytosine and 5-methylcytosine. These two coexisting glycosylases seem redundant for a system targeting mismatched thymine and uracil. Their evolutionary timeline is very different (Figure 4). Both glycosylase ancestors of MBD4 and TDG were found in Bacteria, with twice as many glycosylase orthologs of MBD4 in Bacteria compared to TDG. Glycosylase ancestors of MBD4 were also found in Archaea, whereas only a hypothetical protein TDG Archaea ortholog was identified. Both glycosylase ancestors of MBD4 and TDG were identified in plants and fungi. In contrast, the earliest MBD domain ancestors exist in plants but not in fungi. Most importantly, whereas the TDG protein was found in almost all the species throughout evolution, full length MBD4 emerges as a fusion protein only from Chordates onwards - the representative of Invertebrate-Vertebrate transition. This suggest in a more complex system such as vertebrates, an additional glycosylase like MBD4 is required to maintain genome integrity and MBD4 may possess some special roles such as apoptosis signaling, where fatal mismatches cannot be repaired. Interestingly, according to the evolutionary rate, the proportion of CG sites in vertebrates is far less than that of lower organisms, due to the accumulation of evolutionary repair events of spontaneous deamination. The additional glycosylase system aiming at a lower proportion of CG sites may result in a more precise genome surveillance, which is a requirement in higher animals. It is not surprising that a defect in this system results contributes to disease pathology, for example by increasing the mutation rate at a second site in the two hits (Knudsen) cancer model.

MBD4 is proposed to be a candidate involved in DNA demethylation activity, for example active DNA demethylation at the CYP27B1 promoter in response to PTH (Parathyroid Hormone) exposure [111]. This activity might be stimulated in partnership with DNMT3A and DNMT3B, as the enzymatic excision activity of MBD4 is 30–40-fold lower than its T•G mismatch glycosylase activity [112]. It is interesting that MBD4's glycosylase activity against 5meC can be enhanced by phosphorylation [111]. Similarly TDG was also reported to interact with DNMT3A and DNMT3B and function as a 5meC glycosylase activity against hemimethylated DNA with the same weak excision activity as MBD4 [112]. Furthermore, in Zebrafish, Rai and colleagues showed that MBD4 removes G:T mismatch-specific thymines, which results from 5mC deamination, via the coupling of certain enzymes in the cytidine deaminase family (Activation Induced deaminase (AID) and apolipo-protein B RNA-editing catalytic component (Apobec) [113]. Interestingly, deamination activity by AID/Apobec may not occur unless MBD4 and/or other possible factor are present and/or activated, and a catalytically inactive hMBD4 derivative (D560A) stabilized the putative G:T intermediate and prevented rapid thymine removal [113]. MBD4 was also reported to interact with *Xenopus* DNMT1 in response to DNA damage and DNMT3b [41,83]. Controversially DNMT3b was proposed to methylate cytosine and to deaminate 5-meC, relying on an intrinsic inefficient deaminase activity as part of a gene activation program [83,114,115]. Thymine glycosylases such as TDG and MBD4 may function on deamination of 5-methyl-cytosine by repairing the mismatch.

## 9. Conclusions

The propagation and preservation of epigenetic signatures in development is essential for normal transcription programs. The consequences of loss of these systems can contribute to susceptibility to disease and increased phenotypic variation [17,116]. Loss of this transcriptional ‘dampening’ system is detected by cellular surveillance systems that in many cases can result in activation of intrinsic and extrinsic apoptotic pathways if the epigenetic alterations are detrimental to cell viability.
